# Perceptions and Acceptability of Digital Interventions Among Tuberculosis Patients in Cambodia: Qualitative Study of Video-Based Directly Observed Therapy

**DOI:** 10.2196/16856

**Published:** 2020-07-27

**Authors:** Lila Rabinovich, James Steven Molton, Wei Tsang Ooi, Nicholas Iain Paton, Shelly Batra, Joanne Yoong

**Affiliations:** 1 Center for Economic and Social Research University of Southern California Washington, DC United States; 2 Department of Medicine National University of Singapore Singapore Singapore; 3 Department of Computer Science National University of Singapore Singapore Singapore; 4 Operation ASHA Mumbai India

**Keywords:** directly observed therapy, video recording, telemedicine, mobile health, mHealth, tuberculosis, low-income settings, developing countries, patient acceptance of health care, patient acceptability, Cambodia

## Abstract

**Background:**

Despite the development of effective drugs for treatment, tuberculosis remains one of the leading causes of death from an infectious disease worldwide. One of the greatest challenges to tuberculosis control is patient adherence to treatment. Recent research has shown that video-based directly observed therapy is a feasible and effective approach to supporting treatment adherence in high-income settings. However, few studies have explored the potential for such a solution in a low- or middle-income country setting. Globally, these countries’ rapidly rising rate of mobile penetration suggests that the potential for translation of these results may be high.

**Objective:**

We sought to examine patient perceptions related to the use of mobile health, and specifically video-based directly observed therapy, in a previously unstudied patient demographic: patients with tuberculosis in a low-income country setting (Cambodia).

**Methods:**

We conducted a cross-sectional qualitative study in urban and periurban areas in Cambodia, consisting of 6 focus groups with tuberculosis patients who were receiving treatment (standard directly observed therapy) through a nongovernmental organization.

**Results:**

Familiarity with mobile technology and apps was widespread in this population, and overall willingness to consider a mobile app for video-based directly observed therapy was high. However, we identified potential challenges. First, patients very much valued their frequent in-person interactions with their health care provider, which may be reduced with the video-based directly observed therapy intervention. Second, there may be technical issues to address, including how to make the app suitable for illiterate participants.

**Conclusions:**

While video-based directly observed therapy is a promising technology, even in country settings where mobile penetration is reportedly almost universal, it should be introduced with caution. However, the results were generally promising and yielded important insights that not only will be translated into the further adaptation of key features of video-based directly observed therapy for tuberculosis patients in Cambodia, but also can inform the future design and successful implementation of video-based directly observed therapy interventions in low- and middle-income settings more generally.

## Introduction

### Background

Despite the development of effective drugs for treatment, tuberculosis (TB) remains one of the leading causes of death from an infectious disease worldwide [1]. One of the greatest challenges to TB control is patient adherence to treatment: the need to take drugs daily for 6 months often proves too burdensome, leading to late initiation (treatment delay), disruption (missed treatment), or early termination (treatment default). Nonadherence prolongs the period of infectivity, potentiates relapse, and contributes to the emergence of multidrug-resistant TB cases every year [2].

Conventional directly observed therapy (DOT; the standard TB care whereby patients are strictly monitored daily over the course of their treatment, either by a health care worker or by trained community or family members [3]) faces significant implementation challenges. For patients and their households, adherence entails continuing nonpecuniary costs even when TB drugs themselves are free, including physical side effects, foregone productive time, travel costs, social stigma, and potential affront to autonomy [3]. Reliance on in-person interactions for oversight and support, particularly in resource-poor settings where patients are geographically isolated and hard to reach, is another persistent difficulty.

To address these challenges, numerous adherence-enhancement strategies based on DOT have been tried. Interventions that aim to enhance adherence (eg, reminder systems, defaulter action, education, and peer assistance) without addressing the underlying incentive problem have either shown mixed results or suggested that more high-quality context-specific evidence is needed [4,5]. Studies of financial incentives, however, have shown that patients receiving material rewards are significantly more likely to complete TB treatment [6,7], although these findings were mostly obtained in developed country settings.

With the spread of mobile technology, TB stakeholders are also increasingly eager to explore mobile health (mHealth) solutions to overcoming these constraints [8,9]. The simplest and most widely available approach is the use of text messages or apps to provide support or reminders. However, in the case of TB, use of text message reminders in low- and middle-income country (LMIC) populations has had limited effectiveness [10,11]. More sophisticated interventions, such as smart pills and electronic pill boxes, have been explored and some have shown promise, but practical apps suited to public TB control programs in LMICs are still limited [12-18].

Video-based DOT (VDOT) apps are another mHealth solution that can be integrated into a model of care. By leveraging the ability to take and share videos, VDOT platforms offer a wide range of intervention approaches beyond reminders alone, enhancing monitoring and support by allowing patients to record and send videos of themselves taking medication and enabling new modes of communication with health workers. If successful, such apps would reduce ongoing labor and transport costs by eliminating the need for health workers to travel to patients’ homes and allowing patients to take the drugs daily when convenient, further reducing patient opportunity costs. VDOT has already been shown to be effective in increasing adherence in some high-income country settings [19-25]. However, few studies have explored the potential for such a solution in an LMIC setting; where studies exist (eg, in India, Vietnam, Kenya, and Uganda) they show promise terms of feasibility and acceptability [15,26-29]. Globally, these countries’ rapidly rising rate of mobile penetration suggests that the potential for translation of these results may be high, but at the same time, the speed of such development may result in new and unanticipated challenges.

### Objective

In this context, we sought to examine patient perceptions of issues relevant to the use of mHealth, specifically perceived barriers to treatment adherence, current experience with mobile phone features, and acceptability of VDOT and mobile cash transfers, in a previously unstudied patient demographic: TB patients in a low-income country setting undergoing rapid expansion of mobile penetration. Ultimately, the study aimed specifically to inform the future implementation of an mHealth platform to support TB treatment adherence in this population in Cambodia and, more generally, to highlight considerations when planning and developing such an intervention.

## Methods

### Setting

Our study location, Cambodia, presents a unique opportunity, with a high prevalence of TB, declining TB treatment completion, and increased mobile phone penetration. Approximately two-thirds of all Cambodians carry the TB bacterium, one of the highest rates in the world. DOT can be a challenge for the 80% of the population that lives in rural communities with poor roads and difficult access, served by a mix of health care workers and DOT volunteers. Although Cambodia has maintained outstanding national average treatment success rates of over 90% for well over a decade, disturbing overall trends and subnational disparities have emerged. Since 2009, treatment success and completion rates have declined steadily. Rising rates of drug-resistant TB also indicate that poor adherence may be a concern [30].

Regarding mobile phone penetration, in 2019 there were more mobile phones than people in Cambodia, with more than half the population owning smartphones [7]. This is an increase of 30% over the previous year, a trend that is likely to expand in the lower-income segments of the population, supported by continued declines in prices, as well as the recent introduction of Khmer-language support and system translation for Android version 4.4 and higher (previously available on Nokia and iPhone only). These conditions suggest a large and growing opportunity for mHealth to improve monitoring and enhancement.

### Study Design

We conducted a cross-sectional qualitative study in urban and periurban areas of Cambodia, with the support of Operation ASHA, a nongovernmental organization providing TB treatment and other health services to low-income communities in Cambodia and other Asian countries. Operation ASHA’s community health workers visit patients in their homes every day during the first (intensive) phase of treatment and once weekly during the second (continuous) phase, with patients self-administering the drug the rest of the week.

We conducted 6 focus groups with TB patients being served by Operation ASHA: 3 groups in Phnom Penh (an urban setting) and 3 in Takeo (a semirural province). We determined the number of focus groups using a rule of thumb typically cited in the qualitative literature, namely, that 2 to 5 groups are required per category of participants. Because we had 2 locations (urban and periurban) and for logistical reasons limiting a greater number of focus groups, we settled on 3 groups per location. We recruited 12 participants per group to ensure a minimum of 8 assuming some attrition; ultimately, each group had between 10 and 12 participants. The 2 areas have similar rates of smear-positive results, at around 9% [31].

### Participants

Study participants were men and women aged 18 years and older who had a diagnosis of TB and were currently receiving treatment from Operation ASHA, and who were physically able to travel to Operation ASHA local headquarters. Participants were recruited by Operation ASHA staff using the recruitment script developed by the research team and approved by the institutional review board of the University of Southern California, Los Angeles, CA, USA, which also covers Operation ASHA’s Cambodia work by an agreement of deferral for this study. All participants in the study were assured of confidentiality and anonymity and all identifiers have been removed to protect participant confidentiality. Participants provided oral informed consent, including for audiotaping and transcription, both during recruitment and at the start of the discussion. As a token of appreciation, participants received a US $10 cash incentive for participation. Participants who did not or were unwilling to provide informed consent were excluded from the study.

### Instruments

A semistructured discussion protocol was designed by the lead author with qualitative research expertise (LR) to collect information on patient perceptions of adherence and mHealth interventions in general, as well as to inform the development of a mobile VDOT intervention to support TB treatment adherence. Issues for discussion included perceived barriers to treatment adherence, views on mHealth, and the acceptability of a potential VDOT approach to treatment. The protocol further focused on general issues related to basic features of a VDOT system: (1) sending and receiving text messages, (2) taking and sending videos, and (3) providing incentives via cash or mobile payments as incentives for treatment adherence. We pilot tested the instrument in the first focus group, after which we made minor amendments (primarily clarifying certain questions) for the subsequent groups.

Participants were also asked to assess an existing VDOT app developed by members of the study team (NIJP, JSM, WTO) and asked to evaluate the usability of such an app [32,33]. To avoid priming participants, we presented them with a short description of this VDOT mHealth app only after the general discussion about mobile phone ownership and current use. Users of this platform are provided with a personalized pill schedule that is monitored via the VDOT app. Using the VDOT mobile app, users are able to take a video of themselves taking their medication daily and upload the recorded video for a review by their health worker, together with a report of any adverse effects from the day before. Other features include a color-coded calendar indicating video submission and approval status by day, a function for explaining reasons for missing entries, and a messaging function for communication with the health worker. We also showed participants printouts of what the various screens of the app would look like. We then solicited impressions of the app, including their interest in such an approach, and concerns about its introduction.

We designed a short survey to collect background information prior to the focus group discussion. The questionnaire included questions on demographic and socioeconomic status to provide a complete picture of the background characteristics of our sample. All instruments and the screenshots of the VDOT app were prepared in English, translated into Khmer by bilingual translators, reviewed by an experienced local physician, and further refined where required.

### Data Collection

We conducted all focus groups in July 2018. The focus groups were held in private meeting rooms within Operation ASHA’s health facilities in both study locations and facilitated by a moderator and note taker, both of whom were bilingual native Khmer speakers locally hired and trained in study protocols, facilitation methods, and data capture by 2 study leads (JY and LR). All groups were facilitated in Khmer with only the moderator, note taker, and 1 researcher from the University of Southern California present during the discussion. Discussions lasted approximately 90 minutes and were recorded with permission, transcribed verbatim, and translated into English for analysis.

### Coding and Analysis

We conducted a thematic analysis, in which we coded transcripts using a deductive approach, with codes generated from a review of the transcripts and the study’s research questions and aims. This approach aimed to uncover the key themes and the most significant messages inherent in the raw data [34]. Where needed, the resulting codes were organized into broader, overarching categories. All transcripts were coded by the lead author (LR) using the final version of the codebook. We used the qualitative data analysis software ATLAS.ti version 8.4.4 (1135) (ATLAS.ti Scientific Software Development GmbH) to support the organization, review, and coding of the raw data.

## Results

### Sample Characteristics

We recruited 64 adults receiving TB treatment from Operation ASHA into our study. The sample comprised 34 men and 30 women with a median age of 55 years. The majority (n=44) were married, and 10 were widowed. This is broadly in line with available statistics on TB patients: in the most recently available Cambodian national TB prevalence survey, those aged 45 years or older accounted for 75% of smear-positive TB and 63% of smear-negative, culture-positive TB cases identified, with more males than females having smear-positive and smear-negative, culture-positive TB [35]. Educational attainment was generally low, with 19% (12/64) having no education, 53% (34/64) having an elementary school education, and the rest (18/64, 28%) having either lower or upper secondary education. No one in our sample had any tertiary education. While data are limited on the educational background of TB patients, these numbers show that our sample had overall lower educational attainment than the national population: in 2017, 19% of Cambodians aged 25 years and older reported having no or less than primary education, and 6% reported tertiary education [36]. In our own sample, almost half of participants required assistance from the research staff to complete the paper-based prefocus group questionnaire.

In terms of labor force participation, less than half our sample (29/64) were employed full-time, and 31% were not working at all (20/64). The rest were working part-time (8/64), retired (n=3), or students (n=1). Our sample differed from the general population in this respect: in 2018, labor force participation for all adults aged 15 years and older was estimated at 85% [37]. This difference is likely a function of at least both the median age of our sample (55 years) and the fact that they were all TB patients in treatment.

Regarding TB treatment, 61% (39/64) of our participants had begun their TB treatment less than 4 months previously, with the rest (n=25) in treatment for 5 months or longer.

### Reported Adherence to TB Treatment

In addition to basic sociodemographic information, the questionnaire asked participants about their experience with TB treatment, and their mobile phone ownership and use. The vast majority of our participants reported never having missed any of their medication (55/64, 86%); this is likely because Operation ASHA makes home visits. However, these home visits are not uniformly performed daily [2]: most reported seeing their health worker as part of their TB treatment only once a week (35/64, 55%) or less (n=7, 11%); the rest (n=22, 35%) reported seeing their health worker twice or more a week.

Our focus group discussions revolved around 3 main themes that would inform the rollout of a VDOT intervention in Cambodia: (1) challenges to TB treatment adherence, (2) familiarity with and likely acceptability of features of such a platform, and (3) receptiveness to an mHealth app to support treatment, including use of appropriate cash incentives. We discuss our findings in detail below and provide translated direct quotations to illustrate participants’ views of these different themes.

### Challenges to Treatment Adherence

As expected, participants agreed that access to treatment itself had been easy for them because Operation ASHA provides treatment free of charge and through home visits. Across the groups, participants expressed high levels of satisfaction with the existing Operation ASHA model of home visits:

I speak truly, there is no difficulty [adhering to treatment]. The medicine, they bring it home; [when it] runs out, they bring [more] home.

Female, focus group 5

It was not difficult like before, [when] we took the medicine at the doctor’s place. Now they bring the medicine to our home.

Male, focus group 2

[For] some patients [it is] difficult to travel to the health center, so now we [have] home-based service, it’s [easier] for patients.

Female, focus group 3

Participants tended to highlight the practical benefits of the home visits more than they did the financial benefit of medications for which they did not have to pay out of pocket. Issues of stigma and privacy regarding an individual’s TB and treatment status were not reported as significant concerns, even in the context of receiving a health care worker at home. In addition, there was widespread agreement that home visits not only were more convenient, but also enabled the health care provider to provide the encouragement, reassurance, and support that patients needed to adhere to the treatment, especially in the difficult first 2 months:

The doctor in charge of TB is very helpful. He is very attentive to patients. When I was on treatment he would come in every day to make sure I wouldn’t miss [any dose of] my treatment. I would have missed my treatment if he didn’t push me. After I took the medications I felt so nauseated.

Male, focus group 4

Some [patients] could give up the medicines, so the doctor has to come every day. They travel by motorcycle on difficult road conditions.

Female, focus group 4

While most participants agreed that the no-cost, home-based nature of their TB treatment was a critical factor for accessibility and adherence, they nevertheless recognized that, for themselves and for people they knew, there are significant challenges to treatment adherence, even when the medication is provided at home and at no out-of-pocket cost to the patient.

The main challenges participants identified were physical adverse effects, which can include itching and rashes, nausea, vomiting, lack of appetite, abdominal pain, and fatigue. Across the groups, participants told us of many individuals they knew in their communities who had interrupted treatment because of intolerance to adverse effects:

[Some people] do not want to be healed. They throw away the medicine which cause them some adverse effects.

Male, focus group 1

The reason why patients refuse to take medicines is because of adverse effects. They make them nauseated or feel itchy.

Male, focus group 4

[A neighbor] did not take [the medicine]. He said it’s difficult and [has a] bad smell.

Male, focus group 5

Many also reported that they themselves had experienced difficulties “because there are very terrible adverse effects” (female, focus group 1). Adverse effects, they explained, can be confusing and scary, as in this participant’s experience:

For me after I took the medicines I developed numb joints and the lower part of my body became numb. I was panicked...

Male, focus group 4

Our participants noted that a key difference between nonadherent patients and themselves was that the participants were able to overcome their discomfort with the adverse effects and remain in treatment, as well as their determination to complete their treatment:

When the doctor came here he gave me some medications, which were difficult to swallow for the first 2 months. I had itching, couldn’t stand their strong odor, and felt nauseated. I had so much discomfort for the first 2 months. It was so horrible that I felt I was going to die. My weight then dropped to 56 kg, down from 60 kg. The smell was so repugnant and even if my children cooked porridge and fried or grilled cured dry beef for me I turned them away. But when I finished the larger tablets [and] I began to take the smaller ones I could eat very well and was able to eat anything including pickled vegetables, and my weight now is 63 kg.

Male, focus group 4

We want to be cured, we have to try to be patient with eating, have to think appropriately for taking medicine to be cured. Do not give [away], do not throw [it] out, [we] take it all, and do not leave it.

Female, focus group 5

Another issue participants identified was the pill itself. When asked what may lead a TB patient to interrupt treatment, a few participants agreed that the pills are large and difficult to swallow, which may lead some patients to interrupt treatment very early on. As one participant noted: “Some people complain that the medicine is too big to take” (male, focus group 6). Another one argued that the pill size was especially hard for a particular group: “For elders, [they] cannot swallow” (male, focus group 5). A participant told the group about a neighbor’s experience:

I saw one neighbor, she is a woman. She said when she takes medicine in the first session, the tablets are big. She said when she took medicine, it’s difficult to take it, and [she was] afraid of the medicine. So, she took [it] only for a few days, [then] she gave up, stop taking [the medicine].

Female, focus group 3

A few participants recognized that the length of treatment poses challenges for adherence, even as the majority agreed that following the health care professional’s advice and continuing treatment exactly as prescribed are the key to recovering from the disease. Some admitted that this was true even for themselves, as in the following example:

[W]hen we take [the] medicine, it is difficult and sometime it’s too long time to take. Even I sometime do not want to be cured.

Female, focus group 3

Another participant commented more generally:

[S]ome people take [the medicine] for half the period, and they feel better, then they give up at the half period. They think they are better, they are cured. Actually, this disease, we need time. So, they give up at the half period, they think they are better, they don’t think it will be reactive.

Male, focus group 3

Some participants also expressed the view that there are people with TB who may be “less likely to believe in medicines; they believe in traditional medicine” (male, focus group 1). For example, speaking of a neighbor who had received treatment but not followed it appropriately, a participant said: “Maybe he does not believe. He does not believe that the medicine could treat well” (male, focus group 6).

### Experience With Mobile Phone Features

Most participants (n=41) said they owned a mobile phone, but a significant minority (n=23) reported they did not own one or use a phone owned by a family member. This is well below estimates in 2016 that over 96% of Cambodians aged 18 to 65 years reported owning a mobile phone [7]. A possible explanation for this discrepancy is the higher concentration of older participants in our sample (n=24, 38% individuals ≥60 years old) relative to the national average (estimated at around 7%) [38].

The majority of our participants who owned mobile phones used them for making and receiving phone calls. Other basic functions were far less common. Only 8 people reported using their phones to send or receive text messages, 7 reported using their phones to send or receive money, and only 1 used them to send or receive videos. Nevertheless, while the use of mobile phones to send and receive money was not widespread, many people reported being familiar with Wing (the largest mobile money platform in Cambodia) and other mobile money services when these were brought up in the discussion.

We probed our participants about reasons for not using their phones for money transfers, an element that would allow integration of incentives directly into a mobile app. Those who did not make any mobile cash transfers typically said that it was either because they did not know how to use these apps, or because they had no one to transfer money to or from (or a combination of both these reasons). Among those who had used their mobile phones to send and receive money, the view was that these services were convenient and easy to use:

In terms of using phones to transfer money, I think if we know [how to use it], it’s easier than going to receive [the money] at the bank or organization. It’s easier because the phone is in our hands.

Male, focus group 6

### Receptiveness to VDOT and Cash Incentives

Overall, participants were receptive to the general concept of a mobile app for VDOT. There was widespread recognition that an app could be convenient, as it would save time for both patients (in particular those who still had to attend health facilities for their treatment) and providers:

If we spend time to meet doctor at the health center, it’s difficult. It takes a long time. Sometime, some patients do not have time. So, if you introduce us to use this, it’s easier and saves time.

Female, focus group 3

We can alleviate the doctor’s workload because he would not have to come every day.

Female, focus group 4

At the same time, participants expressed some concerns about this approach. First, some participants worried about the implications of seeing less of their health care provider, since VDOT would replace most of the home visits of the provider. A few individuals across the groups wondered where they would get their medications from if the home visits were discontinued: “If we don’t go to meet the doctor, where can we get the medicine, if he does not bring it for us?” (female, focus group 5).

Second, a related question was whom they would ask questions about their health if their face-to-face interactions with their providers were decreased. Two participants, for example, expressed a clear preference for seeing their health care providers in person rather than having them follow their treatment remotely:

Seeing the doctor in person is better, as she can explain and advise us more.

Male, focus group 4

Go to meet doctor is better...because we can talk face to face for longer discussion.

Male, focus group 5

When presented with the concrete example of the existing VDOT app, there was widespread concern about the technical aspects of the app. Even though the moderator described the app in some detail using printouts with pictures of the various screens and functions, there remained some apprehension among participants that they would not know how to use the app. Nevertheless, there was also agreement that if participants were taught how to use it on their phones they would be willing to try it.

Third, participants who did not own phones at the time of our focus group wondered how the app would apply to them.

In a few instances, participants worried about illiterate patients and how they would be able to navigate an app that, as they saw in the printouts, includes some text.

We also asked participants about their views of cash transfers proposed as incentives to adherence, which could potentially be a component of VDOT platforms. Most people welcomed the incentive idea, but many did not want to comment on how much the incentive should be or said they did not know what would be suitable. Those who agreed to comment on how much would be appropriate argued that somewhere between US $0.50 and US $1 per intake would be acceptable (although a very small number of participants argued for higher incentives, of US $2 or more).

Nevertheless, while our participants generally welcomed a cash incentive, many said that getting the medicines for free was sufficient incentive and money was unnecessary: “Having medicines is good already, sir. There is no need to have cash prize” (female, focus group 4).

## Discussion

### Principal Findings

Our findings showed that familiarity with mobile technology and apps was widespread in this population, and overall willingness to consider a mobile app for VDOT was high. The themes that emerged are in line with VDOT acceptability studies in low-income countries including India, Vietnam, Kenya, and Uganda [15,26-29] and broadly consistent with well-accepted frameworks for assessing the potential acceptability of technology to individuals, such as the technology acceptance model or the unified theory of use and acceptance of technology [39,40].

Our results, however, also highlighted some potential challenges that might arise with transitioning from a face-to-face interaction to VDOT and the resulting implications for interventions. First, many patients related their overall levels of satisfaction with and adherence to frequent, reliable, and high-quality in-person interactions and encouragement. This may then manifest in erosion of both patient satisfaction and adherence in the context of an app-only intervention. In addition, the most frequently cited barriers to adherence were adverse effects, which required physician intervention, and dissatisfaction with the treatment regimen in general, rather than forgetfulness or inconvenience (which are typical factors that work in favor of introducing regular VDOT over periodic in-person visits). Any VDOT intervention may thus need to prioritize timely and frequent feedback and encouragement, as well as ensuring regular interactions with health professionals at intervals, in particular during the first (intensive) phase of treatment, to ensure mitigation of any potential physical and mental harms from less frequent in-person contact.

Second, despite good internet network coverage in our selected areas, not all patients in our study owned a phone. However, almost all patients had access to one. In addition, for most individuals, especially in less urban areas, the use of the phone beyond calls was limited, and familiarity with key aspects of a VDOT platform such as texting and videos was low, with only 1 individual using video regularly. With access to a phone being household-based rather than individual, there may be implications for patient privacy and adherence to a regular schedule of use.

Third, given the rapidly expanding penetration of mobile networks across Cambodia, we expected a higher degree of ownership than we found in our focus group sample, with only 41 individuals personally owning a mobile phone. This may limit the accessibility of an app and lead to concerns such as confidentiality and reliability of access, which may not be assured on a borrowed phone. On the other hand, these findings also suggest that providing phones to participants as part of the intervention may prove a strong incentive to participate. In the focus groups, when the moderator explained that those without phones could be provided with one, enthusiasm and receptiveness to an app significantly increased.

Fourth, the benefits of the app are not likely to accrue to all groups equally. The demographic profile of users may be significant. Younger, more educated, and employed individuals may be most able to use the app and most likely to benefit from a more efficient interaction, whereas older adults may not only find the app more challenging, but also be most likely to experience adverse effects or be averse to losing the in-person interaction. Another issue is how to make the app suitable for illiterate patients. Literacy rates among Cambodian adults have been estimated at around 80%, which makes illiteracy a very significant problem [41]. While the proportion of illiterate TB patients is unclear, it is likely that it will at least mirror, if not exceed, that of the general population. The initial response to the presentation of a specific app platform was relatively subdued among illiterate users. The rollout of such an app may therefore not be universal, and instead would need to take the likely user profile into consideration, with training possibly extended to family members living with or close to the patient, who may act as treatment supporters especially with illiterate or older patients. Future development could focus on designing a language-free interface with visual cues in the form of symbols or icons instead of words.

In spite of these concerns, it is clear from our focus groups that participants were broadly receptive of our proposed innovation, as long as basic safeguards are put in place (in particular phone provision, training, uninterrupted access to the medications, continued access to a health care provider, and suitability for illiterate patients). Moreover, an app was perceived to be convenient not only for themselves; surprisingly, patients felt positive about lessening the burden on providers as well, which is likely to ease a transition to a new platform.

### Limitations

First, one limitation of our study was the reliance on a convenience sample. Individuals who are willing to attend focus groups about adherence may be more likely to be generally adherent or positively disposed toward treatment or Operation ASHA in general.

Second, the use of a hypothetical, graphical presentation of a possible mobile app limited our ability to predict real acceptability and use. This may be especially problematic, as our sample demonstrated remarkably low exposure to apps overall, in spite of network coverage and access to smartphones, potentially due to a lack of familiarity or the lack of relevant service provision via apps at the time of the study. While a more in-depth investigation of factors driving this lack of exposure is beyond the scope of our study, it is worth more detailed exploration. We should reiterate, regardless, that participants were enthusiastic about the possibility of using an app as it was presented to them.

Third, some of our findings are context specific. The level of care provided by ASHA with extensive outreach may not be typical of DOT models in many parts of the world. Furthermore, none of our participants had used mobile apps for TB treatment, let alone health, before. These circumstances may limit broader generalizability across other treatment settings and models of care.

### Conclusion

Our findings suggest that, while VDOT is a promising technology as long as appropriate safeguards are put in place, even in country settings where mobile penetration is reportedly almost universal, it should be introduced with caution. However, the results are generally promising and yield important insights, which not only will be translated into the further adaptation of key features of the VDOT app for TB patients in Cambodia, but also can inform the future design and successful implementation of VDOT interventions in LMIC settings more generally.

## Abbreviations

DOT: directly observed therapy
LMIC: low- and middle-income country
mHealth: mobile health
TB: tuberculosis
VDOT: video-based directly observed therapy
